# Antioxidant Activity of Extracts of *Momordica Foetida* Schumach. et Thonn

**DOI:** 10.3390/molecules18033241

**Published:** 2013-03-13

**Authors:** Rosaria Acquaviva, Claudia Di Giacomo, Luca Vanella, Rosa Santangelo, Valeria Sorrenti, Ignazio Barbagallo, Carlo Genovese, Silvana Mastrojeni, Salvatore Ragusa, Liliana Iauk

**Affiliations:** 1 Department of Drug Science—Biochemistry Section, University of Catania, Catania 95125, Italy; 2 Department of Bio-Medical Sciences, Section of Microbiology, University of Catania, Catania 95125, Italy; 3 Department of Health Sciences, University “Magna Graecia” of Catanzaro, Catanzaro 88100, Italy

**Keywords:** *Momordica foetida* Schumach. et Thonn., antioxidant activity, adipogenic differentiation

## Abstract

*Momordica foetida* Schumach. et Thonn. (Cucurbitaceae) is a perennial climbing herb with tendrils, found in swampy areas in Central Uganda. Antidiabetic and antilipogenic activities were reported for some *Momordica* species, however the mechanism of action is still unknown. Oxidative stress may represent an important pathogenic mechanism in obesity-associated metabolic syndrome. The present study evaluated free radical scavenging capacity of different concentrations of aqueous, methanolic and dichloromethane leaf extracts of *Momordica foetida* Schumach. et Thonn. and the ability of these extracts to inhibit *in vitro* plasma lipid peroxidation; in addition, healthy human adipose mesenchymal stem cell cultures were used in order to test the hypothesis that these extracts may affect adipocyte differentiation. Results obtained in this study suggested that aqueous extract might be useful in preventing metabolic syndrome.

## 1. Introduction

*Momordica foetida* Schumach. et Thonn. (Cucurbitaceae) is a medicinal plant, widely distributed in tropical Africa, south and west tropical Africa, that has both male and female flowers [1]. It is a perennial climbing herb with tendrils, the flowers are cream colored, often with a reddish or orange centre and its characteristic fruit is bright orange with prickles. *M. foetida* Schumach. et Thonn. is found in swampy areas in Central Uganda. Drinking of aqueous leaf extracts of the plant to treat malaria is reported in East and Central Africa [2]. Other medicinal uses of extracts of this plant include the treatment of hypertension, peptic ulcers, diabetes mellitus, and as a purgative [2,3]. Curcubitane triterpenoids, polyphenolic compounds, have been isolated from leaf extracts, and alkaloids and glycosides from whole plant extracts. Antidiabetic and antilipogenic activities were also reported for some *Momordica* species, however the mechanism of action is still unknown [4].

Several studies have correlated obesity with high levels of lipid peroxidation and decreased antioxidant levels [5,6,7]. Oxidative stress, which results when free-radical formation exceeds protective antioxidant mechanisms or the later are compromised, has become a focus of intense interest in most biomedical disciplines and many types of clinical research; increasing evidence from research show that oxidative stress is associated with the pathogenesis of obesity and it has been demonstrated that *in vitro* pre-adipocyte proliferation and differentiation can be controlled by redox metabolism [8,9,10,11,12,13,14,15,16,17] suggesting that reactive oxygen species (ROS) are involved in adipocyte differentiation.

In the present study the free radical scavenging capacity of different concentrations of aqueous, methanolic and dichloromethane leaf extracts of *Momordica foetida* Schumach. et Thonn. was evaluated by *in vitro* assays; moreover the ability of these extracts to inhibit plasma lipid peroxidation *in vitro* was also evaluated. In addition, human adipose mesenchymal stem cell (hMSC) cultures were used in order to test the hypothesis that these extracts may also affect adipocyte differentiation.

## 2. Results and Discussion

Table 1 reports the total phenolic and flavonoid contents of the three different extracts. Aqueous extract resulted richer in phenolic and flavonoid compounds compared to methanol and dichloromethane extracts.

**Table 1:** Total polyphenols and total flavonoids in three different extracts of leaves of *Momordica foetida* Schumach. et Thonn.

Extract	Total Phenolic content μM Gallic acid	Total Flavonoid Content μM Catechin
Aqueous	50 ± 0.05	2.80 ± 0.20
Methanolic	15 ± 0.01	4 ± 0.09
Dichloromethane	5 ± 0.01	0.3 ± 0.03

**Figure 1:**
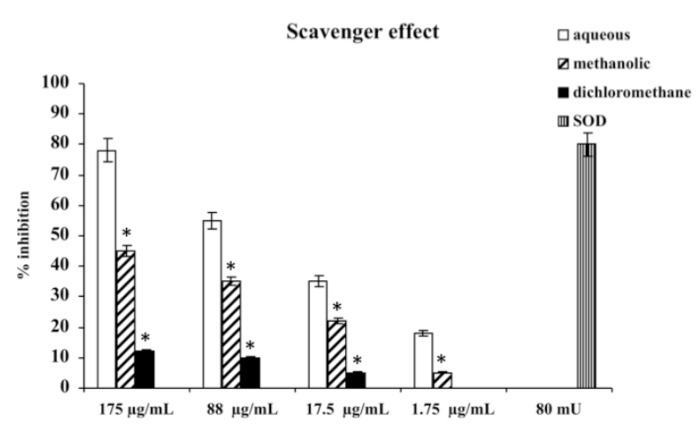
Scavenger effect of extracts of *M. foetida* Schumach. et Thonn. on superoxide anion; results are expressed as percentage of inhibition of NADH oxidation (rate of superoxide anion production was 4 nmol/min). Each value represents the mean ± SD of five experimental determinations. * *p* < 0.001 *vs.* the same concentration of the aqueous extract.

Consistent with their different polyphenol contents, scavenger activities of the three extracts differed, depending on the type of extract. The aqueous extract has proven the most effective scavenger, with an effect that, at 175 μg/mL, was comparable with 80 mU superoxide dismutase (SOD). The methanolic and dichloromethane extracts exhibited scavenger activities lower than aqueous; the less active was dichloromethane extract (Figure 1).

**Figure 2:**
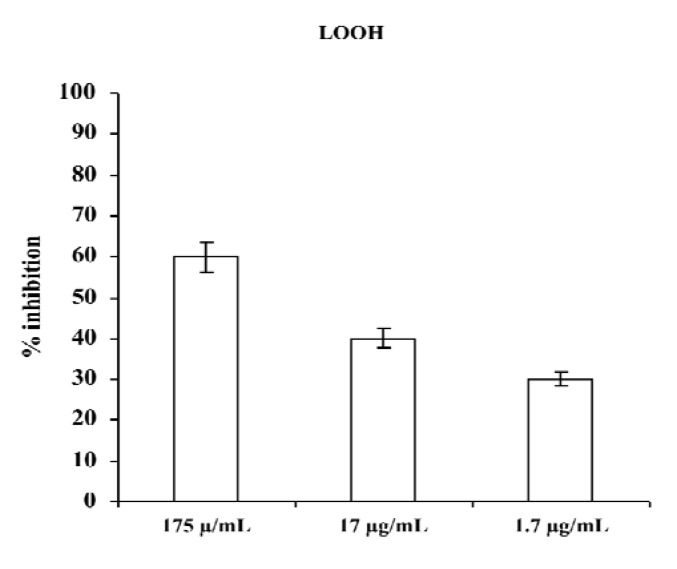
Effect of aqueous extract of *M. foetida* Schumach. et Thonn. on LOOH production in plasma; results are expressed as percentage of inhibition of LOOH formation with respect to the same sample incubated in absence of the extract. Each value represents the mean ± SD of five experimental determinations.

Based on polyphenol contents and our results regarding scavenger activities of the three extracts, aqueous extract was chosen for subsequent experiments. Figure 2 shows results obtained by incubating plasma of a healthy donor in the presence of different concentrations of aqueous extract of *M. foetida* Schumach. et Thonn.; as seen, the presence of the extract during incubation of plasma caused a considerable and dose-dependent inhibition of plasma lipid hydroperoxide (LOOH) formation.

These results demonstrated that antioxidants present in aqueous extract of leaves of *M. foetida* Schumach. et Thonn. are able to counteract radical chain reactions, preventing peroxidative damage of plasma lipids beyond the action of antioxidants naturally present in plasma.

We also tested the hypothesis that the aqueous extract of *M. foetida* Schumach. et Thonn. might affect adipogenic differentiation of human adipose mesenchimal cells. These cells are capable of differentiating into many different types of phenotypes, including osteoblasts, myocytes, chondrocytes and adipocytes *in vivo* and *in vitro* [18,19,20]. The directed differentiation of MSC can be performed *in vitro* using the appropriate media and the adipogenic differentiation is confirmed by specific staining.

**Figure 3:**
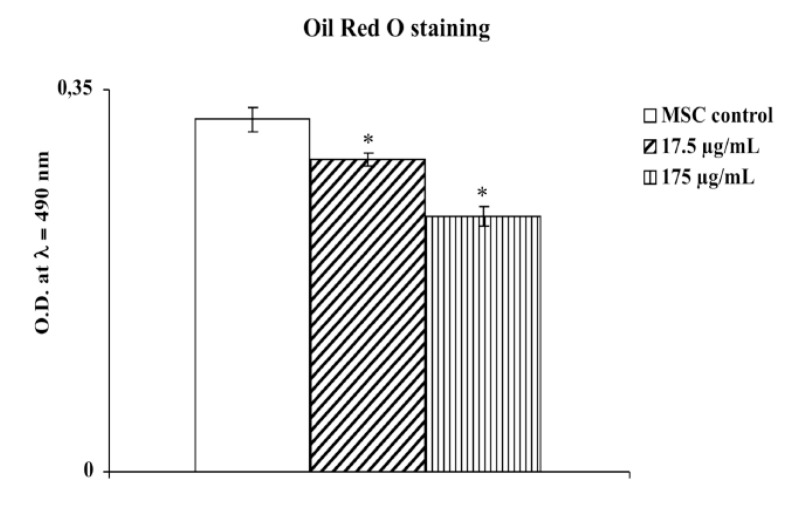
Effect of aqueous extract of leaves of *M. foetida* Schumach. et Thonn. on adipogenesis of hMSCs as measured by Oil Red O staining. Each value represents the mean ± S.D. of five experimental determinations. * *p* < 0.001 *vs.* control.

As seen in Figure 3, our results demonstrated an increase in adipogenesis and accumulation of lipid droplets in cultured human adipose tissue-derived mesenchimal cells compared to the same cells treated with the aqueous extract of *M. foetida* Schumach. et Thonn.

It has been suggested that increased levels of reactive oxygen species (ROS), with consequent shifting of the intracellular redox status *vs.* oxidant conditions, promote adipogenesis [9,11,13,15]. Then, the decreased adipogenesis observed in hMSC cultured in the presence of aqueous extract of *M. foetida* Schumach. et Thonn., might be ascribed to its free radical scavenger effects; in order to verify this hypothesis, ROS were determined using a fluorescent probe, 2',7'-dichlorofluorescein diacetate (DCFH-DA). After diffusion into the cells, this probe is deacetylated by cellular esterases to a non-fluorescent compound which is oxidized by ROS into 2',7'-dichlorofluorescein (DCF), a highly fluorescent compound whose fluorescence intensity is proportional to the levels of ROS [21].

**Figure 4:**
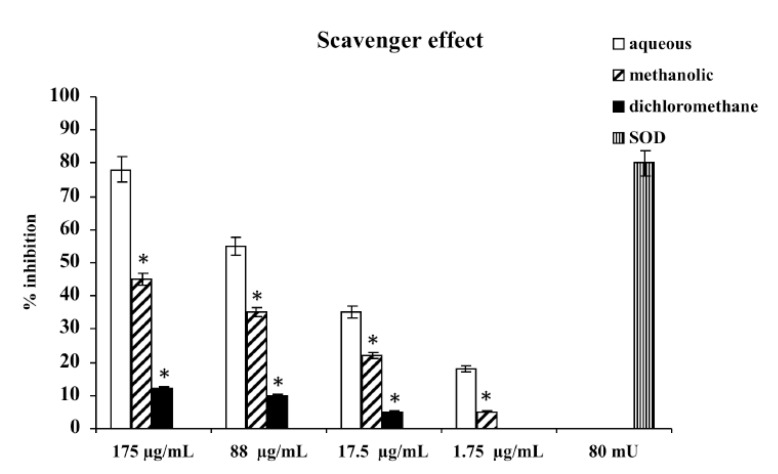
Determination of ROS by DCFH method in cultured hMSC: effect of aqueous extract of *M. foetida* Schumach. et Thonn. Each value represents the mean ± S.D. of five experimental determinations. * *p* < 0.001 *vs.* the same cells cultured in the absence of plant extract (control). Results are expressed as Fluorescence Intensity (F.I.) × 1 × 10^6^/mg prot).

As reported in Figure 4, the exposure for 72 h of hMSCs to 17.5 µg/mL or 175 µg/mL aqueous extract of *M. foetida* Schumach. et Thonn. resulted in a significant decrease in ROS. These data confirm the results obtained using *in vitro* cell-free systems and further support our hypothesis that antiadipogenic activity of *M. foetida* Schumach. et Thonn. might be due to a decrease in intracellular ROS levels.

## 3. Experimental

### 3.1. Chemicals

Water, methanol and dichloromethane used for the extractions were of analytical grade and were purchased from Merck S.P.A. (Milan, Italy); all the other solvents, chemicals and reference compounds were purchased from Sigma-Aldrich (Milan, Italy).

### 3.2. Plant Collection and Preparation of Extracts

The leaves of *Momordica foetida* Schumach. et Thonn. were collected in August 2010 in the Eastern Democratic Republic of Congo and kindly provided by Sister Kavira Muhyama, Diocese of Butembo-Beni, Bunyuka. A voucher specimen of the plant was deposited in the herbarium of Department of Health Sciences, University “Magna Graecia” of Catanzaro. Aqueous, methanolic and dichloromethane extracts were obtained by maceration of each powdered plant sample (2.5 g) in water, methanol and dichloromethane (50 mL), respectively, for 24 h under constant shaking at room temperature. The extracts were filtered and evaporated to dryness under reduced pressure with a rotatory evaporator.

### 3.3. Total Phenolic and Flavonoid Content

The concentration of total phenolic compounds was determined spectrophotometrically, using the Folin-Ciocalteu total phenols procedure, as described by Ballard *et al*. [22], with modifications. Known amounts of gallic acid were used to prepare the standard curve. Appropriately diluted test extracts (0.1 mL) and the gallic acid standard solutions (0.1 mL) were transferred to 15 mL test tubes. Folin-Ciocalteu reagent (3.0 mL, 0.2 N) was added to each test tube and the contents mixed using a vortex mixer. After 1 min, 9.0% (w/v) Na_2_CO_3_ in water (2.0 mL) was added and the solution was mixed. Absorbance was determined at λ = 765 nm. The concentration of total phenolic compounds in the extracts was determined comparing the absorbance between the extract samples and the gallic acid standard solutions. All samples were determined in triplicate. Total phenolic content was expressed as µmol gallic acid/L ± S.D.

The flavonoid concentration was measured using a colorimetric assay [23], with modifications. A standard curve of cathechin was used for quantification. Briefly, aqueous, methanolic and dichloromethane extracts (25 mL) and/or cathechin standard solutions were added to H_2_O (100 mL). At time zero, 5% NaNO_2_ (7.5 mL) was added; after 5 min, 10% AlCl_3_ (7.5 mL) was added and at 6 min, 1 M NaOH (50 mL) was added. Each reaction mixture was then immediately diluted with H_2_O (60 mL) and mixed. Absorbances of the mixtures upon the development of pink color were determined a λ = 510 nm relative to a prepared blank. The total flavonoid contents of the samples are expressed as μmol catechin/L. Each result represents the mean ± S.D. of three experimental determinations.

### 3.4. Scavenger Effect on Superoxide Anion

Superoxide anion was generated *in vitro* as described by Acquaviva *et al*. [24]. A total volume of 1 mL of the assay mixture contained 100 mM triethanolamine-diethanolamine buffer, pH 7.4, 3 mM NADH, 25 mM/12.5 mM EDTA/MnCl_2_, 10 mM β-mercaptoethanol; samples contained different concentrations (1.75-17.5-88-175 μg/mL) of the three (aqueous, methanolic and dichloromethane) extracts of leaves of *M. foetida* Schumach. et Thonn. After 20 min incubation at 25 °C, the decrease in absorbance at λ = 340 nm was measured. Results are expressed as percentage of inhibition of NADH oxidation. SOD (80 mU) was used as reference compound. Each result represents the mean ± S.D. of five experimental determinations.

### 3.5. Determination of Lipid Hydroperoxide Levels in the Plasma of a Healthy Donor

Heparinized venous blood of a healthy volunteer donor, who agreed to take part in the study and gave his written consent, was collected after overnight fasting. Plasma was separated by centrifugation at 800 *g* for 20 min. Plasmatic lipid hydroperoxide levels were evaluated by oxidation of Fe^2+^ to Fe^3+^ in the presence of xylenol orange at λ = 560 nm [25]. Heparinized venous blood was collected after overnight fasting; plasma was separated by centrifugation at 800 *g* for 20 min. Plasma aliquots (500 μL) were diluted 1:1 with oxygenated PBS and incubated at 37 °C for 2 h with or without different concentrations of the aqueous extracts (1.75-17.5-175 μg/mL) in a total volume of 1 mL. Results are expressed as percentage of inhibition respect to control (plasma incubated in absence of test compounds) and represent the mean ± S.D. of five experimental determinations.

### 3.6. Isolation and Adipogenic Differentiation of Human Adipose MSCs

Adipose tissue sample was obtained from a patient underwent abdominal plastic surgery (male, 25 years old); the subject provided his written consent before inclusion in the study, which was conducted according to the guidelines of the Ethical Commitee of the University of Catania, Italy. Adipose tissue sample was removed under sterile conditions, washed in PBS, minced, and digested with 1 mg/mL collagenase type I in 0.1% BSA for 1 h at 37 °C in a shaking water bath. The pellet was collected by centrifugation at 650 *g* for 10 min and then treated with red blood cell lysis buffer (155 mM NH_4_Cl, 10 mM KHCO_3_ and 0.1 mM EDTA) for 10 min at room temperature. After centrifugation the cellular pellet was filtered through a 100-μm mesh filter to remove debris. The filtrate was centrifuged, and the obtained stromal vascular fraction was plated onto 100 mm cell culture dishes in complete culture medium (DMEM containing 20% fetal bovine serum, 100 μg/mL streptomycin, 100 U/mL penicillin, 2 mM L-glutamine, and 1 μg/mL amphotericin-B). Cells were cultured at 37 °C in humidified atmosphere with 5% CO_2_. After 24 h, non-adherent cells were removed, and adherent cells were washed twice with PBS. Confluent cells were trypsinized and expanded in T75 flasks (passage 1). A confluent and homogeneous fibroblast-like cell population was obtained after 2–3 weeks of culture. For all the experiments, only cells at early passages were used. At 50–60% confluence the medium was replaced with adipogenic medium, and the cells were cultured for an additional 15 days. The adipogenic media consisted of complete culture medium supplemented with DMEM-high glucose (4.5 g/L), 10% (w/v) FBS, 10 mg/mL insulin, 0.5 mM dexamethasone and 0.1 mM indomethacin. Media were changed every 2 days.

### 3.7. Oil Red O Staining and Lipid Droplet Size

Oil Red O staining is an assay performed to stain induced adipogenic cultures to detect mature adipocytes. The histological mechanism of the staining of lipids is invariably a function of the physical properties of the dye being more soluble in the lipid than in the vehicular solvent. The polyazo group of dyes include the oil red series, the Sudan red series and the Sudan blacks.

For Oil Red O staining, 0.21% Oil Red O in 100% isopropanol was used. Briefly, MSC-derived adipocytes, after 15 days, were fixed in 10% formaldehyde, washed in Oil-red O for 10 min, rinsed with 60% isopropanol, and the Oil red O eluted by adding 100% isopropanol for 10 min and Optical Density measured at λ = 490 nm in a microplate reader (Synergy HT multi-mode microplate reader, BioTek, Milan, Italy). Results are expressed as optical density (OD) at λ = 490 nm. Each result represents the mean ± S.D. of five experimental determinations.

### 3.8. Determination of ROS

Determination of ROS was performed by using the fluorescent probe DCFH-DA, as previously described [26]. Briefly, 100 μL of 100 μM DCFH-DA, dissolved in 100% methanol, was added to the cellular medium, and cells were incubated at 37 °C for 30 minutes. After incubation, cells were lysed and centrifuged at 10,000 × *g* for 10 min. The fluorescence (corresponding to oxidized DCF) was monitored spectrofluorometrically (λ_ex_ = 488 nm; λ_em_ = 525 nm), using an F-2000 spectrofluorimeter (Hitachi, Tokyo, Japan) and results were expressed as Fluorescence Intensity (F.I.) × 1 × 10^6^/mg protein. Total protein content in each sample was evaluated according to Lowry *et al*. [27].

### 3.9. Statistical Analysis

One-way analysis of variance (ANOVA) followed by Bonferroni’s *t* test was performed in order to estimate significant differences among samples. Data were reported as mean values ± S.D. and differences between groups were considered to be significant at *p* < 0.005.

## 4. Conclusions

Results reported in the present study demostrate that the aqueous extract of *Momordica foetida* Schumach. et Thonn. has antioxidant activity that can be ascribed to its high content in phenolic and flavonoid compounds. Moreover it is also able to inhibit adipogenesis of hMSC, probably due to its anti-radical activity. These data confirm the therapeutic uses of *Momordica foetida* Schumach. et Thonn. aqueous extract and also suggest that it might be a useful tool in preventing metabolic syndrome.
